# Genome-Wide Detection of CNVs and Association With Body Weight in Sheep Based on 600K SNP Arrays

**DOI:** 10.3389/fgene.2020.00558

**Published:** 2020-06-09

**Authors:** Zhipeng Wang, Jing Guo, Yuanyuan Guo, Yonglin Yang, Teng Teng, Qian Yu, Tao Wang, Meng Zhou, Qiusi Zhu, Wenwen Wang, Qin Zhang, Hua Yang

**Affiliations:** ^1^College of Animal Science and Technology, Northeast Agricultural University, Harbin, China; ^2^Key Laboratory of Animal Genetics, Breeding and Reproduction, Education Department of Heilongjiang Province, Harbin, China; ^3^State Key Laboratory of Sheep Genetic Improvement and Healthy Production, Xinjiang Academy of Agricultural and Reclamation Science, Shihezi, China; ^4^Institute of Animal Nutrition, Northeast Agricultural University, Harbin, China; ^5^Department of Animal Genetics and Breeding, College of Animal Science and Technology, Shandong Agricultural University, Tai’an, China

**Keywords:** body weight, copy number variation, sheep, SNP, breed-specific

## Abstract

Copy number variations (CNVs) are important genomic structural variations and can give rise to significant phenotypic diversity. Herein, we used high-density 600K SNP arrays to detect CNVs in two synthetic lines of sheep (DS and SHH) and in Hu sheep (a local Chinese breed). A total of 919 CNV regions (CNVRs) were detected with a total length of 48.17 Mb, accounting for 1.96% of the sheep genome. These CNVRs consisted of 730 gains, 102 losses, and 87 complex CNVRs. These CNVRs were significantly enriched in the segmental duplication (SD) region. A CNVR-based cluster analysis of the three breeds revealed that the DS and SHH breeds share a close genetic relationship. Functional analysis revealed that some genes in these CNVRs were also significantly enriched in the olfactory transduction pathway (oas04740), including members of the OR gene family such as *OR6C76*, *OR4Q2*, and *OR4K14*. Using association analyses and previous gene annotations, we determined that a subset of identified genes was likely to be associated with body weight, including *FOXF2*, *MAPK12*, *MAP3K11*, *STRBP*, and *C14orf132*. Together, these results offer valuable information that will guide future efforts to explore the genetic basis for body weight in sheep.

## Introduction

Copy number variations (CNVs) are key structural variations wherein DNA segments between 1 kilobase and several megabases in length undergo duplication or deletion, thereby giving rise to substantial genetic variation (Feuk et al., 2006). CNVs can cause changes in traits or diseases by affecting gene structure or dosage (Zhang et al., 2009). CNVs are widespread, accounting for 4.8–9.5% of the human genome (Zarrei et al., 2015). Certain CNVs have been associated with many diseases and complex traits in human, such as obesity (Turner et al., 2015), BMI (Willer et al., 2008; Macé et al., 2017), and body weight (Willer et al., 2008; Macé et al., 2017). Some CNVs additionally impact phenotypic variation in domestic species, altering traits such as coat color in horse, pigs, and sheep (Rosengren Pielberg et al., 2008; Fontanesi et al., 2011b; Rubin et al., 2012); production traits in cattle (Seroussi et al., 2010); and reproductive traits in pigs and cattle (Sironen et al., 2006; Pei et al., 2019).

Recent high-throughput sequencing studies have facilitated the genome-wide detection of CNVs in sheep (Fontanesi et al., 2011a; Liu et al., 2013; Hou et al., 2015; Ma et al., 2015, 2017; Jenkins et al., 2016; Zhu et al., 2016; Yang et al., 2018; Di Gerlando et al., 2019b), goats (Fontanesi et al., 2010; Liu et al., 2019b), cattle (Liu et al., 2010, 2019a), pigs (Wang et al., 2017, 2019), horses (Ghosh et al., 2014; Kader et al., 2016), chickens (Rao et al., 2016; Gorla et al., 2017), dogs (Alvarez and Akey, 2012; Di Gerlando et al., 2019a), and rabbits (Fontanesi et al., 2012). The first sheep CNV map was constructed by Fontanesi et al. (2011a) using a tiling oligonucleotide array with ∼385,000 probes that had been designed using the bovine genome for reference. More recently, several studies based upon SNP genotyping platforms and array-based comparative genomic hybridization (aCGH) have identified ubiquitous genetic variants within the sheep genome.

A subset of studies has focused on genome-wide CNV identification efforts in different sheep breeds. For example, Zhu et al. (2016) identified 371, 301, and 66 CNVRs in large-tailed Han, Altay, and Tibetan sheep, respectively. Similarly, Ma et al. (2017) detected 1296 CNV regions (CNVRs) in Chinese Tan sheep, while Di Gerlando et al. (2019b) identified 365 CNVRs in Valle del Belice sheep. Work by Yang et al. (2018) detected population differences in CNVs among different breeds of sheep across geographical regions, with clear lineage-specific CNVRs being detectable within diverse breeds, thus offering insight into breed-specific population histories.

Some studies (Liu et al., 2010; Wang et al., 2013) have suggested that the construction of an accurate ovine CNV map will necessitate surveying multiple populations from differing genetic backgrounds as a means of validating previously identified CNVRs and allowing for more reliable CNV mapping. In this study, two synthetic sheep lines (DS and SHH sheep) and Hu sheep (a local Chinese sheep breed) were selected for CNV mapping using a high-density Affymetrix 600K genotyping platform. This study additionally sought to explore the functional characteristics of these CNVs through gene, QTL, GO, and KEGG annotation analyses. To further understand the genetic basis of sheep productive traits, we performed an association study to identify CNVs related to birth body weight (BIRTH_WT), weaning body weight (WEAN_WT), and yearling body weight (BW).

## Materials and Methods

### Population Selection and SNP Genotyping

For this study, a total of 40 Hu sheep (a highly fecund breed of sheep native to China), 165 DS sheep (a synthetic line from the progressive hybridization of Australian Suffolk sheep and Chinese Hu sheep), and 65 SHH sheep (a cross breed between DS sheep and Chinese Kazakh sheep) were collected from the Xinjiang Academy of Agricultural and Reclamation Science.

Genomic DNA was extracted from the ear tissue of these sheep using a conventional phenol/chloroform extraction method. Whole genomic DNA from 270 individual samples was genotyped using the Affymetrix Ovis600K Genotyping BeadChip according to provided instructions. We developed the quality control filter criteria used for SNP identification in this study. First, those SNPs that mapped to the sex chromosomes or failed to map were excluded. Second, individuals and SNPs with a call rate ≤95% were discarded. Third, those SNPs with a minor allele frequency (MAF) <1% were discounted. A total of 467,502 autosomal SNP markers and 270 sheep were used for CNV detection.

### Genome-Wide CNV Detection

A hidden Markov model was used to detect autosomal CNVs with PennCNV^1^. After CNV detection, PennCNV quality control was performed with the following cutoffs: log R ratio (LRR) standard deviation < 0.3, B allele frequency (BAF) drift <0.01, and a waviness factor between −0.05 and 0.05, with each CNV including 3+ consecutive SNPs. According to the definition of CNVs proposed by Feuk et al. (2006), those with a CNV length ≤1 kb were discarded. After quality control, 10 sheep were discarded.

### CNVR Map Construction

CNV regions were identified via aggregating overlapping CNVs from all samples, based upon the criteria defined by Zhou et al. (2016). To further improve the reliability of the results, all CNVs that were called only once in the population were discarded. We then divided CNVRs into gains, losses, and complex CNVRs (including gain and loss events). In this study, a CNV map was constructed based on the *Ovis aries* (OAR3.1) genome assembly. To investigate the relationship between the numbers of CNVRs located on each chromosome and length of the chromosome, a regression analysis was performed using the R language.

CNV frequencies within a given CNVR were assessed and used to compare the three breeds of sheep analyzed in this study. CNV frequencies (CNV count within each CNVR/sample count within each CNVR) in each individual breed were estimated, and variance across breeds was calculated. Based on CNVR frequencies across three breeds, Euclidian distances were calculated. Using Ward’s method as the linkage criteria, a hierarchical clustering analysis was performed using 45 CNVR at top 5% of the variances of frequency. This process was performed using the R pheatmap package.

There have been eight studies related to the genome-wide identification of sheep CNVs. Of these, there are 3 previous studies based on the OAR1.0 genome assembly, with all other studies being based on the OAR3.1 genome assembly. Those CNVRs that were mapped on the OAR1.0 assembly were therefore converted to the OAR3.1 assembly format in order to facilitate a more accurate comparison. Coordinates of these CNVRs were converted using NCBI Remap^2^.

### Annotation Analysis

BioMart^3^ in the Ensemble database was used to identify those genes which overlapped with CNVRs. Functional Gene Ontology (GO) and Kyoto Encyclopedia of Genes and Genomes (KEGG) analyses of these genes were performed using DAVID^4^. Furthermore, sheep quantitative trait loci (QTLs) were identified using the Animal QTL database^5^. We used chi-squared analyses to inspect the relationship between CNVRs and the segmental duplication (SD) region of the sheep genome, based upon the results of Feng et al. (2017).

### qPCR Validation of CNVRs

To confirm the accuracy of identified CNVRs, 14 CNVRs were selected randomly from among all detected CNVRs. For each of these CNVRs, we selected animals predicted by PennCNV to have different status of CNVs (Loss, Gain, or Complex) for the validation experiment. Together with three other sheep predicted by the PennCNV to be normal, a total of 52 sheep were used. PCR was then conducted using FastStart Universal SYBR Green Master Mix on the QuantStudio 6 Flex detection system. The Primer Premier 5.0 software was used for primer design based on the NCBI reference sequences (Supplementary Table S4). The sheep *DGAT1* gene was used as a reference gene in this study. Three samples predicted to be normal by PennCNV were used as reference samples. The 2^–ΔΔ*CT*^ method was used to quantify the copy number, and the relative quantification (RQ) value was calculated. Samples with RQ values below 0.59 (ln1.5) denote copy number loss individuals; samples with RQ values about 1.59 (ln3) or more denote copy number gain individuals (more three copies).

### CNVR Association Analyses

We have measured BIRTH_WT (*n* = 218), WEAN_WT (*n* = 165), and BW (*n* = 194) for the experimental sheep population. We selected 20 CNVRs that had been detected in at least 10% of the samples, for an association analysis between CNVRs and body weight. For the CNV association study, the statistical model used was as follows: *y_*ijk*_* = μ + *t_*i*_* + *b_*j*_* + *c_*k*_* + *e*_*ijk*_, where *y* is the phenotypic observation, μ is the population mean, *t*_*i*_ is the year effect, *b*_*j*_ is the breed effect, *c*_*k*_ is the CNV effect, and *e*_*ijk*_ is the random residual vector. In this study, we considered CNV effects to be binary (present or absent). For the association analysis of WEAN_WT and BW traits, the BIRTH_WT trait was added to the model as a covariate. Using the SAS GLM process, we performed a CNV association analysis for each trait.

## Results

### Genome-Wide Detection of CNVs

**FIGURE 1:**
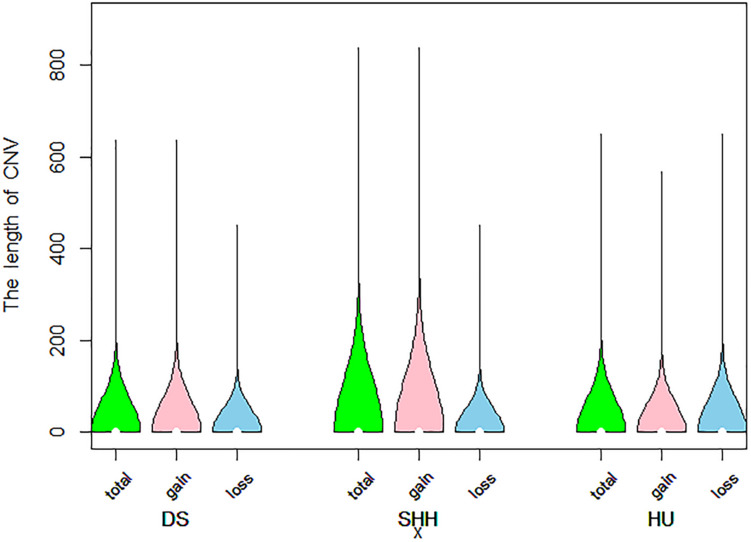
Violin plots of the total CNV lengths, gain CNV lengths, and loss CNV lengths in each sheep breed.

A total of 9103 CNVs were detected in our analysis of on sheep autosomes, including 7,394 copy number gains and 1,709 copy number losses (see Table 1). Lengths of these CNVs ranged from 1 to 839.20 kb, with approximately 83.8% of these CNVs being less than 50 kb long. On average, the CNV number of individuals was 35, overlapping 1.15 Mb region of the genome. The length of these CNVs is different in different breeds. The length of these CNVs ranged from 1.00 to 635.92 kb, 1.01 to 839.20 kb, and 1.00 to 649.27 kb in DS, SHH, and Hu sheep, respectively. The distribution of CNV sizes is shown in Figure 1.

**TABLE 1:** Summary of CNVs identified in three sheep populations.

Breed	Samples	CNVs	Gain events	Loss events	Total length (Mb)	Average length each CNV (kb)
DS	162	5316	4336	980	163.34	30.73
SHH	64	2352	1950	402	79.13	33.65
Hu	34	1435	1108	327	55.97	39.01
Total	260	9103	7394	1709	298.45	32.79

### Genome-Wide Sheep CNVR Characteristics

Overlapping CNVs were merged into non-redundant CNVRs. A total of 919 CNVRs were detected in these three breeds (Supplementary Table S1), consisting of 730 gains, 102 losses, and 87 complex CNVRs (copy number gain and copy number loss events within the same region). We detected more gain than loss events, and these gains had slightly larger average sizes than did losses (48.13 kb vs. 39.49 kb).

**FIGURE 2:**
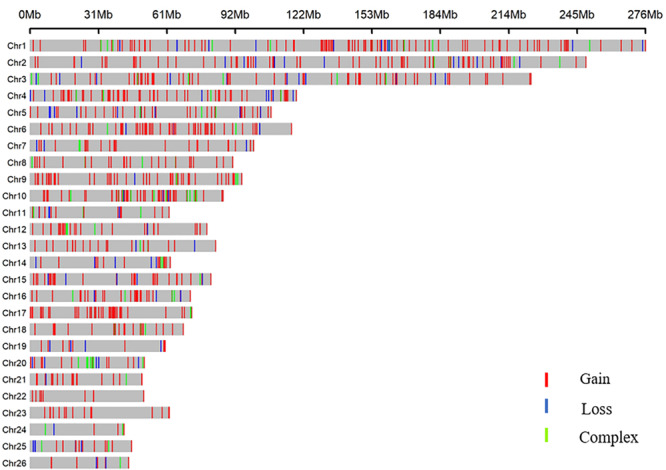
CNVR distributions in the genomes of three sheep breeds.

**FIGURE 3:**
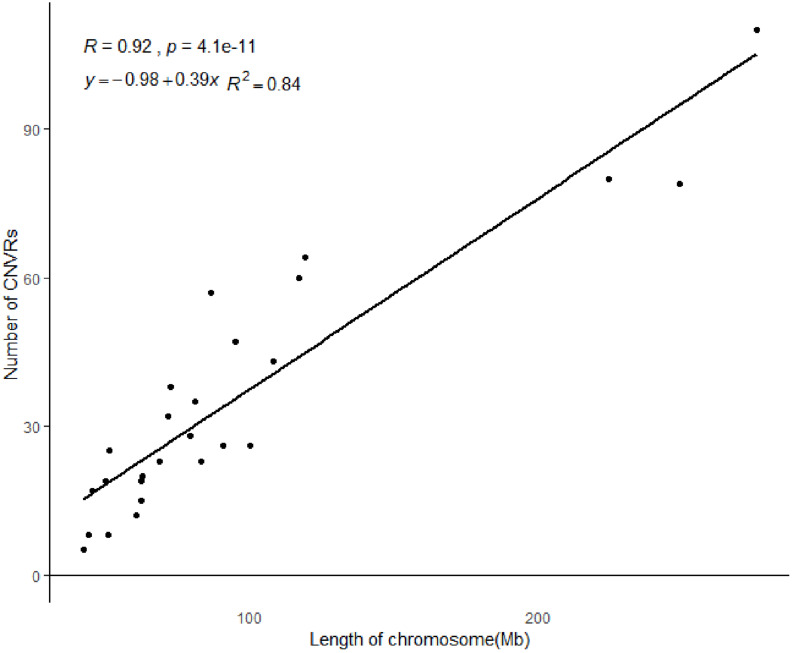
The correlation between CNVR numbers and chromosome length.

All 919 CNVRs correspond to 1.96% of the sheep genome (48.17 Mb/2452.07 Mb). Figure 2 summarizes the locations and characteristics of all CNVRs in the genome. These CNVRs were unevenly distributed among different chromosomes. Chromosome 1 harbored the greatest number (110) of CNVRs, while chromosome 10 had the greatest CNVR density with an average distance of 1516.62 kb between CNVRs. Regression analysis revealed a significant positive linear relationship between chromosome length and the number of CNVRs located on that chromosome (*R*^2^ = 0.84, *P*-value = 4.1E-11) (Figure 3), such that longer chromosomes contained more CNVRs.

Distribution plots indicated the presence of certain CNVR hotspots in the sheep genome. Segmental duplication (SD) has been shown to be a necessary condition and catalyst for the formation of genome CNVs in many mammals and has increasingly been a focus of genetic variation research (Liu et al., 2009). In this study, we found that 13.63 Mb of the 48.17-Mb CNVRs directly overlapped with SDs. Through a chi-squared test, we found that sheep CNVRs were significantly enriched in the SD region (*P* = 5.27E-19).

**FIGURE 4:**
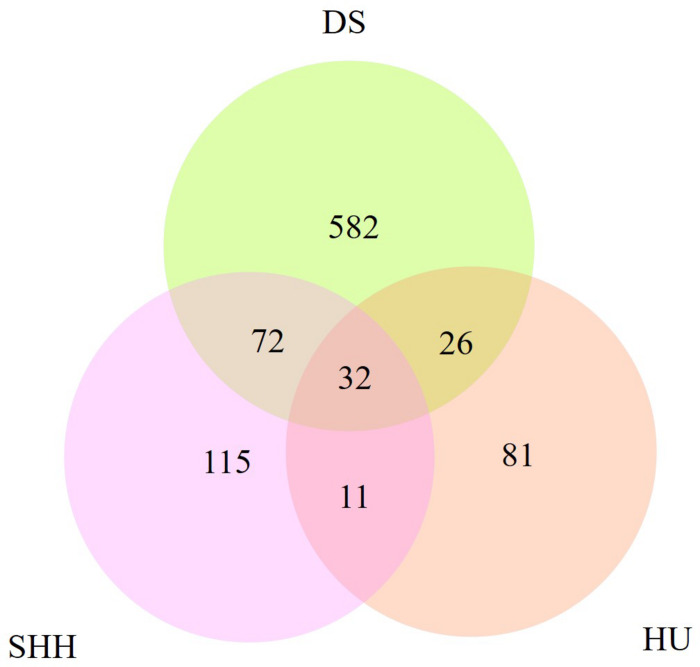
The number of CNVRs identified in these sheep breeds and the number of CNVRs overlapping between breeds.

Table 2 summarizes the genome-wide CNVR events from each sheep population. There were 582, 115, and 81 CNVRs detected only in DS, SHH, and Hu sheep, respectively, while 32 CNVRs were detected in all three breeds, as shown in Figure 4. These results indicated that the number of CNVR events differed among breeds, which may be due to the different genetic backgrounds of these populations or the different samples taken for each breed. We treat CNVRs only in one breed as breed-specific CNVRs.

**TABLE 2:** Summary of CNVRs identified in three sheep populations.

Breed	CNVR	Total length (Mb)	Average length (kb)	Gain type	Loss type	Complex type	Percentage covered genome CNVRs (%)
DS	712	36.24	50.90	566	73	73	1.48%
SHH	230	13.75	59.78	158	35	37	0.56%
Hu	150	9.81	65.40	85	32	33	0.40%
Total	919	48.17	52.41	730	102	87	1.96%

**FIGURE 5:**
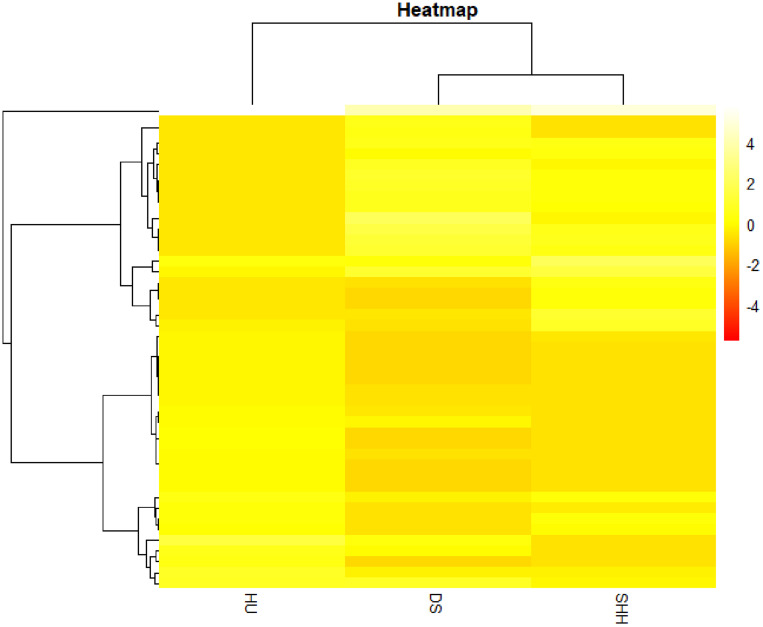
Hierarchical clustering-based heat map analysis.

In addition, we estimated the variance of each CNVR frequency among three breeds and selected the CNVRs of the top largest 5% variance for cluster analyses. The results of this analysis revealed that these CNVRs could distinguish the three breeds in this study from one another (see Figure 5). DS sheep and SHH sheep were preferentially clustered into one group and were then clustered with Hu sheep. This cluster structure is consistent with the breeding history and bloodline relationship of these three sheep breeds.

### Annotation Analysis

Genes overlapping with identified CNVRs were identified and annotated using OARv3.1 from the BioMart system in Ensemble^6^. This analysis indicated that 391 CNVRs (42.55%) overlapped with 688 genes, including 585 protein-coding genes, 84 lincRNAs, and 19 microRNAs (Supplementary Table S2). GO and KEGG pathway analyses were next conducted to gain insight into the functional roles of these genes. Following Bonferroni correction, two molecular function terms (GO:0004984, olfactory receptor activity; GO:0004930, G-protein coupled receptor activity) and one KEGG pathway (oas04740, Olfactory transduction) were found to be significantly enriched (see Table 3).

**TABLE 3:** GO and KEGG pathway analyses of genes in the identified CNVRs.

Category	Term	GO name	Count	*P*-value	Bonferroni
Molecular function	GO:0004984	Olfactory receptor activity	36	1.87E-14	3.17E-12
Molecular function	GO:0004930	G-protein coupled receptor activity	39	9.28E-13	1.58E-10
Cellular component	GO:0016021	Integral component of membrane	121	1.34E-09	2.41E-07
Cellular component	GO:0005886	Plasma membrane	51	6.00E-05	1.07E-02
KEGG_PATHWAY	oas04740	Olfactory transduction	42	8.71E-06	1.42E-03

A total of 482 QTLs associated with different traits overlapped with sheep CNVRs (Supplementary Table S3). Among these QTLs, there were 164, 108, 72, 80, 21, 20, and 17 related to the meat and carcass trait, the health trait, the production trait, the milk trait, the exterior trait, the wool trait, and the reproduction trait, respectively.

### qPCR Validation of CNVRs

**FIGURE 6:**
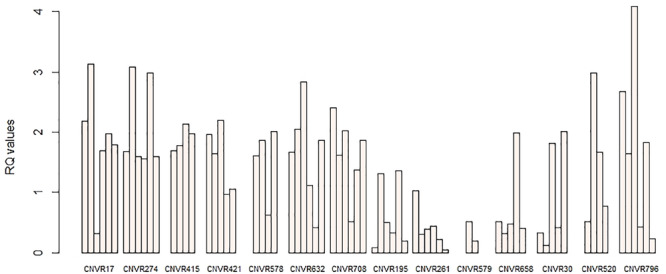
qPCR validation of selected CNVRs. The *y*-axis shows the RQ values obtained by qPCR, while the *x*-axis indicates the sample names in the different CNV regions. Samples with RQ values of about 1 denote normal individuals (two copies); samples with RQ values below 0.59 (ln1.5) denote copy number losses; samples with RQ values of about 1.59 (ln3) or more denote copy number gains (>three copies).

In order to confirm the accuracy of our CNVR predictions, we randomly selected 14 CNVRs to validate via qPCR. These CNVRs were selected from all three breeds and represented different predicted types of CNVs (gains, losses) (Table 4). We performed 58 qPCR assays in 52 sheep. Overall, 87.93% (51) of these chosen CNVRs were successfully confirmed in agreement with the predictions made by PennCNV. Validation results are shown in Figure 6.

**TABLE 4:** qPCR validation results.

CNVR ID	Position	Detected type by PennCNV	Validated type	Validated
CNVR17	OAR1:48.84–49.00	Gain	Gain	Yes
CNVR274	OAR4:13.89–13.92	Gain	Gain	Yes
CNVR415	OAR6:71.00–71.03	Gain	Gain	Yes
CNVR421	OAR6:78.35–78.57	Gain	Gain	Yes
CNVR578	OAR10:61.82–61.88	Gain	Gain	Yes
CNVR632	OAR12:52.23–52.40	Gain	Gain/loss	Yes
CNVR708	OAR15:56.86–56.94	Gain	Gain/loss	Yes
CNVR195	OAR3:13.16–13.23	Loss	Loss	Yes
CNVR261	OAR3:185.84–185.87	Loss	Loss	Yes
CNVR579	OAR10:61.98–62.05	Loss	Loss	Yes
CNVR658	OAR13:52.75–52.77	Loss	Loss/gain	Yes
CNVR30	OAR1:81.56–81.66	Complex (gain/loss)	Gain/loss	Yes
CNVR520	OAR9:65.60–65.64	Complex (gain/loss)	Gain/loss	Yes
CNVR796	OAR18:37.82–37.89	Complex (gain/loss)	Gain/loss	Yes

### CNV Association Analyses

The descriptive statistics for each trait are summarized in Table 5. In total, the average ± S.D. (standard deviation) of BIRTH_WT, WEAN_WT, and BW were 3.22 kg ± 0.88, 33.25 kg ± 9.40, and 54.39 kg ± 13.12, respectively. Twenty CNVRs were selected for association analysis. Among those CNVRs, CNVR586 (OAR10: 70.34–71.40 Mb) and CNVR338 (OAR5: 9.08–9.14 Mb) were the most frequently detected (69.23%) and the least frequently detected (10.00%), respectively. We determined that CNVR367 and CNVR747 were significantly associated with weaning body weight and yearling body weight, respectively, using a linear regression model (Table 6). On the basis of the online sheep QTL database, we determined that CNVR747 overlapped with QTL #14305 (associated dressing percentage) and QTL #14272 (related to lean meat yield percentage), while CNVR367 overlapped with QTL #12934 and QTL #17204, associated with body weight (birth) and meat palmitoleic acid content, respectively.

**TABLE 5:** Descriptive statistics for each trait.

Trait	Individuals	Mean (kg)	Standard deviation (kg)	Minimum (kg)	Maximum (kg)
BIRTH_WT	218	3.22	0.88	1.40	6.20
WEAN_WT	165	33.25	9.40	12.50	64.00
BW	194	54.39	13.12	25.00	87.00

*Traits include birth body weight (BIRTH_WT), weaning body weight (WEAN_WT), and yearling body weight (BW).*

**TABLE 6:** Genome-wide association analysis of the relationship between CNVs and phenotypic.

CNVR ID	Position	Type	*P*-value		
			BIRTH_WT	WEAN_WT	BW
CNVR6	OAR1:31.57–31.68	Gain	0.3067	0.6782	0.2857
CNVR129	OAR2:87.36–87.39	Loss	0.4968	0.4697	0.9208
CNVR178	OAR2:210.87–210.93	Loss	0.2896	0.2161	0.1770
CNVR239	OAR3:137.5–137.6	Complex	0.1016	0.6644	0.0896
CNVR314	OAR4:83.01–83.25	Gain	0.5591	0.6974	0.9339
CNVR328	OAR4:112.3–112.65	Complex	0.9765	0.6360	0.8036
CNVR338	OAR5:9.08–9.14	Loss	0.9180	0.8957	0.6708
CNVR349	OAR5:39.18–39.3	Loss	0.4132	0.2389	0.9642
CNVR367	OAR5:83.15–83.24	Complex	0.1920	0.0141*	0.6858
CNVR464	OAR8:2.21–2.44	Gain	0.7407	0.6965	0.6238
CNVR535	OAR9:92.92–92.97	Complex	0.4648	0.9534	0.3978
CNVR556	OAR10:41.61–41.74	Complex	0.2591	0.9788	0.7926
CNVR569	OAR10:55.44–55.5	Complex	0.8679	0.9379	0.8870
CNVR586	OAR10:70.34–71.4	Complex	0.2244	0.5882	0.9852
CNVR587	OAR10:71.62–72.25	Complex	0.4881	0.4347	0.1200
CNVR678	OAR14:58.59–58.74	Complex	0.4558	0.8033	0.8884
CNVR688	OAR15:7.98–7.99	Loss	0.2854	0.6090	0.2283
CNVR747	OAR16:64.54–64.65	Complex	0.5969	0.5708	0.0333*
CNVR835	OAR20:26.96–27.06	Complex	0.5224	0.2376	0.9929
CNVR847	OAR20:50.42–50.62	Complex	0.1460	0.4487	0.7094

Traits included birth body weight (BIRTH_WT), weaning body weight (WEAN_WT), and yearling body weight (BW). *P value < 0.05.

## Discussion

Copy number variation has been increasingly recognized as an important source of genetic variation and may be one of the main contributors to phenotypic diversity and evolutionary adaptation in animals. Non-allelic homologous recombination (NAHR) between low copy repeats or segmental duplications is a major mutational mechanism thought to be responsible for CNV generation. Some studies suggest that segmental duplication may promote CNV formation in primates, goats, and sheep (Perry et al., 2006, 2008; Dumas et al., 2007; Lee et al., 2008; Fontanesi et al., 2009, 2010). In this study, we found that 1/3 of identified CNVRs were also enriched in the SD regions. Results obtained by Hou et al. (2011) indicated that 1/4 of cattle CNVRs mapped to segmental duplications with a total overlap of about 16 Mb. CNVs are known to co-occur with SDs, with some studies suggesting that CNVs represent polymorphic drifting SDs that have become fixed within the genome (Freeman et al., 2006; Goidts et al., 2006; Perry et al., 2006; Sharp et al., 2006; Kim et al., 2008).

In this study, more than 50% CNVRs were detected only in DS, SHH, and Hu sheep, as shown in Figure 4. Liu et al. (2010) similarly detected breed-specific copy number differences in different cattle breeds, indicating that some cattle CNVs are likely to arise independently in breeds and to contribute to differences between these breeds. To highlight the potential evolutionary contributions of these CNVs to sheep breed formation and adaptation, we generated a heat map for the 45 CNVRs with the greatest frequency differences in our analyses. This hierarchical clustering plot indicated that DS and SHH sheep are more closely related, which is consistent with known breed divergence and history. So we deem that some CNVRs may be breed-specific or breed-differential (see Table 7), due to altered metabolic requirement due to the herd environment, feeding mode, breeding methods, and the reproductive strategy through human selection. These CNVRs are likely to arise independently in different breeds and to contribute to sheep domestication and breed formation. Of note, the observed CNV frequency differences between breeds may be the result of both selection and genetic drift arising due to genetic bottlenecks for certain breeds. So, some CNVRs have the potential to offer insight into the characteristics of that breed, pending further studies of the phenotypic effects of these CNVs.

**TABLE 7:** Some CNVRs contribution to differences between these breeds.

	Breed	CNVR ID	Location	Type	Candidate genes
Breed-specific	DS	19	1:51.50–51.53	Gain	*ASB17*
	DS	275	4:14.71–14.76	Gain	
	DS	287	4:32.20–32.51	Gain	*DBF4*, *CCDC126*, *ABCB1*
	DS	353	5:49.60–49.68	Complex	*PCDHB6*, *PCDHB7*
	DS	354	5:49.70–49.71	Loss	*PCDHB14*
	DS	384	6:26.11–26.19	Gain	*RAP1GDS1*
	DS	566	10:50.84–50.87	Gain	*TBC1D4*
	DS	599	11:8.67–8.68	Loss	*OR4D2*
	DS	706	15:55.30–55.32	Gain	*ANO3*
	DS	837	20:28.00–28.00	Gain	*ZFP57*, *MOG*
	Hu	189	2:244.55–244.60	Gain	*EIF4G3*
	Hu	233	3:115.06–115.11	Loss	*SYT1*
	Hu	241	3:142.71–142.76	Gain	*TWF1*, *IRAK4*
	Hu	255	3:164.24–164.46	Gain	
	Hu	261	3:185.84–185.87	Loss	
	Hu	430	6:90.76–90.94	Gain	*STBD1*, *CCDC158*
	Hu	480	8:57.46–57.56	Gain	*MOXD1*
	Hu	510	9:46.76–46.76	Loss	*NCOA2*
	Hu	733	16:42.57–42.76	Complex	
	Hu	824	20:0.99–1.01	Loss	*KHDRBS2*
	SHH	85	1:187.24–187.26	Gain	*KALRN*
	SHH	111	2:2.28–2.31	Gain	*RAB14*, *CNTRL*
	SHH	283	4:20.69–20.73	Complex	*SCIN*
	SHH	329	4:113.87–113.90	Gain	*GALNTL5*
	SHH	394	6:42.86–42.98	Loss	
	SHH	511	9:50.73–50.83	Gain	
	SHH	541	10:13.94–13.97	Gain	*ENOX1*
	SHH	670	14:38.06–38.12	Loss	
	SHH	671	14:42.15–42.21	Gain	*ZNF507*, *DPY19L3*
	SHH	717	15:77.13–77.27	Loss	*OR5M11*, *OR5AR1*, *OR5M10*
Breed-different	DS, Hu	442	7:21.93–22.18	Complex	*TRDV3*, *TRDC*, *TRDV2*, *TRAV41*
	DS, Hu	617	12:13.24–13.41	Gain	*BRINP3*
	DS, Hu	836	20:27.70–27.85	Complex	*RPF2*, *TRIM26*
	DS, Hu	839	20:28.38–28.52	mixed	*OR2I1P*
	DS, Hu	847	20:50.42–50.62	Complex	*FOXF2*
	DS, SHH	556	10:41.61–41.74	Complex	
	DS, SHH	632	12:52.23–52.40	Gain	*PRAMEF2*
	DS, Hu, SHH	328	4:112.30–112.65	Complex	*GIMAP5*, *GIMAP1*, *GIMAP4*
	DS, Hu, SHH	338	5:9.08–9.14	Loss	*ADGRE2*
	DS, Hu, SHH	586	10:70.34–71.40	Complex	*ERVW-1*
	DS, Hu, SHH	587	10:71.62–72.25	Complex	

We investigated function of genes encompassing these breed-specific or breed-differential CNVRs (see Table 7). Our findings reveal that some genes related to immunity and defense (such as *CNTRL*, *IRAK*, *MOG*, *RAP1GDS1*, *SCIN*, and *TRDV3*), neurological system processes (such as *BRINP3*, *ENOX1*, *KALRN*, *PCDHB14*, *PCDH15*, and *SYT1*), sensory perception (such as *CCDC126*, *KHDRBS2*, *MOXD1*, *OR2I1P*, *OR5AR1*, *OR5M10*, and *OR5M11*), lipid metabolic development (such as *NCOA2*), muscle development (such as *ANO3* and *TBC1D4*), and reproduction procession (such as *ASB17*, *DPY19L3*, *EIF4G3*, and *GALNTL5*).

We compared the results of the present analysis to previously identified sheep CNVRs (Liu et al., 2013; Hou et al., 2015; Ma et al., 2015, 2017; Jenkins et al., 2016; Zhu et al., 2016; Yang et al., 2018; Di Gerlando et al., 2019b). Of the 919 CNVRs detected herein, 357 (38.85%) partially or wholly overlapped with previously reported CNVRs (Table 8). This suggests that roughly 40% of the CNVRs that we detected have been previously validated, while the remaining 60% are novel. It is important to note that only a small proportion of CNVRs identified in our study overlapped with those found in other studies. Similar findings were also observed in CNV studies conducted in humans and other mammals (Wang et al., 2014; Letaief et al., 2017). These inconsistencies may be a result of differences in the detection platforms or algorithms used in the corresponding analyses, due to variations in the genetic backgrounds of analyzed sheep, differences in study population in size and structure, or random or technical errors in certain analyses. This also suggests that many CNVs that exist within the sheep genome have yet to be discovered.

**TABLE 8:** Comparison of CNVRs identified in this and prior studies.

Study	Platform	Sample	Number of CNVRs	Total length (Mb)	Average length (kb)	Range (kb)	Gain	Loss	Complex	Genomic	Concordant number with our study
Liu et al., 2013	50K SNP	329	238	60.35	253.57	14–1296	13	219	6	OARv1.0	55 (23.11%)
Hou et al., 2015	CGH	5	51	15.55	304.10	52–2093	23	21	7	OARv1.0	27 (52.94%)
Ma et al., 2015	50K SNP	160	111	13.75	123.78	14–567	12	99	0	OARv3.1	3 (2.70%)
Jenkins et al., 2016	CGH	30	3488	67.60	19.38	1–3550	1325	2023	140	OARv3.1	61 (1.75%)
Zhu et al., 2016	600K SNP	110	490	81.04	165.39	100–804	93	390	7	OARv3.1	112 (22.86%)
Ma et al., 2017	600K SNP	48	1296	121.80	92.70	1–2344	118	1173	5	OARv3.1	87 (6.71%)
Yang et al., 2018	50K SNP	2111	619	196.94	318.15	14–4631	–	–	–	OARv1.0	33 (5.33%)
Di Gerlando et al., 2019b	50K SNP	416	365	118.36	348.10	17–1818	43	320	2	OARv3.1	79 (21.64%)
This study	600K SNP	260	919	48.17	52.41	1–1069	730	102	87	OARv3.1	–

We additionally summarized the detailed characteristics of sheep CNVRs reported in prior studies (Table 8). In general, the length of CNVRs identified based on the 50K SNP chip is much longer than those based on the HD SNP chip and the CGH array. This CNV size difference is likely due to sampling differences or to variations in resolution and genome coverage between these techniques. For example, the SNP chip resolution (mean probe spacing) was 50 and 4 kb for the 50-kb SNP chip and the 600-kb SNP chip, respectively, whereas that of the aCGH platform was 1.2 and 1.8 kb in studies conducted by Hou et al. (2015) and Jenkins et al. (2016), respectively. This indicates that the CGH array provides an advantage over the SNP chip for CNV detection, as it is able to reveal the presence of many small CNVs in addition to large ones. This may explain why the largest number of CNVRs was identified in a study conducted by Jenkins et al. (2016), with only 1.7% (61/3844) of these CNVRs being consistent with our findings. As such, future experiments employing high-throughput sequencing methods have the potential to remedy these differences by allowing for the identification of much shorter CNVRs. Gene ontology analyses have revealed that CNVRs are particularly enriched in genes related to immunity, sensory perception (e.g., smell, sight, and taste), responses to external stimuli, and neuro-developmental processes (reviewed in De Smith et al., 2008). Some GO terms related to immunity or neuro-developmental processes were not found to be enriched in our study following Bonferroni correction. Relevant genes enriched in the olfactory receptor pathway include members of the olfactory receptor (OR) gene family, such as *OR6C76*, *OR4Q2*, *OR4K14*, *OR8K1*, *OR5M11*, and *OR5AR1*, as reported in other CNV studies of German Mutton, Dorper, and Sunite sheep (Liu et al., 2013). Odors are essential for animal survival as they enable animals to locate food, to detect predators or environmental toxins, and to select mates (Spehr and Munger, 2009). Olfactory receptors are also thought to have an additional role in appetite regulation. ORs constitute the largest gene family in the mammalian genome. These ORs are G-coupled protein receptors with a 7-transmembrane structure and are responsible for triggering the olfactory signal transduction pathway (Young et al., 2008). In the human genome, some human ORs exhibit high copy numbers due to segmental duplications (Bailey et al., 2001). Previous human CNVR studies have found many of these regions to contain genes in the OR family (Sebat et al., 2004; Tuzun et al., 2005; Conrad et al., 2006). Variations in OR repertoires among species have been shown to be a result of duplication and deletion events following species divergence (Young et al., 2002; Quignon et al., 2005; Niimura and Nei, 2007). Paudel et al. (2015) found that the majority of CNV genes in the genus Sus are OR genes that are important for mate identification and foraging activities. As such, these authors hypothesized that high rates of OR CNV variability allow species to rapidly adapt to specific environments, making these genes particularly important for Sus speciation activities.

Based upon our enrichment analyses, association analyses, and the known functions of identified genes, we highlighted certain genes of interest that overlapped with CNVRs in this study, including *FOXF2*, *MAPK12*, *MAP3K11*, *STRBP*, and *C14orf132*. The following serves as a summary of the basic functions of these genes (shown in Table 9). *FOXF2* encodes fork-head box F2. The human *FOXF2* gene is associated with three M syndrome (Linhares et al., 2015), which results in short stature and abnormal facial features as a consequence of abnormal skeletal growth. Changes in *FOXF2* copy number may lead to the occurrence of congenital diaphragmatic hernia (Yu et al., 2012). The *MAPK12* gene (mitogen-activated protein kinase 12) is known to be of particular importance during myotube differentiation, playing key roles in regulating myogenic precursor cell proliferation in the context of muscle growth and regeneration. *MAP3K11* is a serine/threonine kinase gene that positively regulates the FGFR signaling pathway, which plays an important role in the control of cartilage and bone formation (Montero et al., 2000). *STRBP* (spermatid perinuclear RNA-binding protein) is involved in spermatogenesis and sperm function and plays a role in regulating cell growth and movement (Gallardo-Arrieta et al., 2010). The *C14orf132* gene is a large intergenic lincRNA. Through CNV and transcriptomic analyses, Tiirats et al. (2016) found *C14orf132* to be potentially related to an extremely low birth weight phenotype.

**TABLE 9:** Candidate genes overlapping with CNVs.

Gene symbol	Location (Mb)	Full name	Function of association with
*FOXF2*	OAR20:50.50–50.50	Forkhead box F2	Related to abnormal skeletal growth. Lead to the occurrence of congenital diaphragmatic hernia.
*MAPK12*	OAR3:223.86–223.87	Mitogen-activated protein kinase 12	Related to muscle growth and regeneration.
*MAP3K11*	OAR21:43.09–43.11	Mitogen-activated protein kinase kinase kinase 11	Related to the control of cartilage and bone formation.
*STRBP*	OAR3:12.50–12.56	Spermatid perinuclear RNA binding protein	Related to spermatogenesis and regulating cell growth and cell movement.
*C14orf132*	OAR18:59.64–59.64	Chromosome 14 open reading frame 132	Related to an extremely low birth weight phenotype.

Due to the high conservation of genes between humans and sheep, genes that are known to be related to complex human traits may also be important for related traits in sheep. However, further research will be needed to formally test the functional relevance of these genes.

## Conclusion

In this study, we performed CNV detection using a 600K SNP array on 260 individuals from three breeds of sheep (DS, SHH, and Hu), leading us to identify a total of 919 CNVRs from these populations. Together, these results serve to supplement extant CNVR map information. In an association analysis exploring the relationship between CNVRs and body weight traits, we found that CNVR367 and CNVR747 were significantly associated with weaning body weight and yearling body weight, respectively. In addition, in an analysis of CNVR overlapping genes, we identified additional genes that may be related to body weight traits, including *FOXF2*, *MAPK12*, *MAP3K11*, *STRBP*, and *C14orf132*. Our results offer meaningful genomic insights that will help to guide future research and to provide a preliminary basis for the future exploration of the relationship between CNVs and body weight traits.

## Data Availability Statement

The variation data reported in this article have been deposited in the Genome Variation Map (GVM) in Big Data Center, Beijing Institute of Genomics (BIG), and Chinese Academy of Sciences, under accession numbers GVM000068 that are publicly accessible at https://bigd.big.ac.cn/gvm/getProjectDetail?project=GVM000068. The Bioproject accession number is PRJCA002639.

## Ethics Statement

The guidelines for the Care and Use of Laboratory Animals were carefully followed during this study, which received approval from the Experimental Animal Care and Use Committee of Xinjiang Academy of Agricultural and Reclamation Sciences (Shihezi, China, approval number: XJNKKXY-AEP-039, January 22, 2012). All procedures and animal collections were also approved by the Northeast Agricultural University (Harbin, China) Animal Care and Treatment Committee (IACUCNEAU20150616). Written informed consent was obtained from the owners for the participation of their animals in this study.

## Author Contributions

HY, ZW, and JG conceived the study. HY, ZW, JG, and YG participated in its design. YY, QY, WW, and HY were involved in the acquisition of data. JG performed all data analysis. HY, ZW, and JG drafted the manuscript. YG, YY, TT, TW, MZ, QiuZ, WW, QinZ, and QY contributed to the writing and editing. All authors read and approved the final manuscript.

## Conflict of Interest

The authors declare that the research was conducted in the absence of any commercial or financial relationships that could be construed as a potential conflict of interest.

## Footnotes

1 http://penncnv.openbioinformatics.org/en/latest/

2 https://www.ncbi.nlm.nih.gov/genome/tools/remap

3 http://asia.ensembl.org/biomart/martview/

4 https://david.ncifcrf.gov/

5 http://www.animalgenome.org/cgi-bin/QTLdb/OA/browse

6 http://asia.ensembl.org/biomart/martview/

## Supplementary Material

TABLE S1 The detailed of CNVRs on autosomes.

TABLE S2 Gene list overlapped with CNVR.

TABLE S3 QTL list overlapped with CNVR.

TABLE S4 The primer for qPCR.
